# A Study on the Evolution of Subsequent Yield Surfaces for Ductile Cast Iron Under Different Pre-Deformation Loading Conditions

**DOI:** 10.3390/ma19102089

**Published:** 2026-05-16

**Authors:** Shihong Huang, Shenghuan Qin, Jie Pang

**Affiliations:** Department of Management Science and Engineering, Guangxi University of Finance and Economics, Nanning 530007, China; huangshihong1234@gxufe.edu.cn (S.H.); 2012210030@gxufe.edu.cn (J.P.)

**Keywords:** ductile cast iron, subsequent yield surface, pre-deformation

## Abstract

This study experimentally investigates the subsequent yield surfaces of thin-walled tubular ductile cast iron (QT600-7) specimens under various pre-deformation histories and elucidates their evolution patterns. The effects of pre-deformation level, unloading position, and loading path on the subsequent yield surfaces are examined, with particular attention to the concave phenomenon observed in the measured yield surfaces. The results show that the subsequent yield surfaces of QT600-7 translate towards the pre-loading direction. Translation and distortion are more pronounced at smaller offset strains and gradually diminish with increasing offset strains. Under different pre-loading paths, a sharp corner appears in the pre-loading direction and a concave or flattened shape forms in the opposite direction when the offset strain is small; this concave phenomenon tends to disappear under larger offset strains. A higher pre-deformation level leads to more noticeable distortion of the yield surface corresponding to small strains. After pre-tension and unloading, if reverse compression occurs, the subsequent yield surface under small offset strains exhibits a complex shape in the opposite direction.

## 1. Introduction

The failure of engineering structures ultimately stems from material failure in local components, and most materials undergo significant plastic deformation prior to failure. The plastic behavior of materials is highly complex, primarily characterized by nonlinear stress–strain responses after yielding, which depend strongly on the loading history. Classical plasticity theory primarily comprises three key components: the yield criterion, the plastic flow rule, and the hardening law. The yield surface defines the boundary between elastic and plastic deformation in stress space. Its initial shape and its evolution during plastic deformation—known as the subsequent yield surface—directly determine the plastic flow, hardening behavior, and eventual failure mode of materials under complex loading paths, such as cyclic or non-proportional loading. Therefore, research on the yield surface and the evolution of subsequent yield surfaces is essential.

The establishment of plasticity constitutive theory relies on a foundation of experimental research, and the refinement and development of constitutive models require experimental validation. However, existing studies have not yet established a consistent framework describing yield surface evolution laws [1,2,3,4,5,6]. Experiments have demonstrated that the position and shape of a material’s subsequent yield surface in stress space exhibit anisotropic characteristics during the loading process due to plastic deformation and the Bauschinger effect. For instance, Ohashi et al. [7] applied combined axial load and torque to thin-walled tubular specimens of mild steel to investigate the anisotropic plastic behavior exhibited by initially isotropic materials due to deformation history and developed an analytical model to describe the observed anisotropic plastic behavior. Cheng et al. [8] used strain control to test thin-walled tubular specimens of an Al/Mg alloy and found that subsequent yield surfaces undergo translation, changes in shape and size, and exhibit a cross effect. Kuwabara et al. [9] employed a servo-controlled tension-internal pressure testing machine to conduct a series of experimental studies on the anisotropic plastic deformation behavior of aluminum alloy tube A5154-H112, establishing that both yield functions considered were effective phenomenological plasticity models for predicting the anisotropic plastic deformation behavior of the material. Helling et al. [10] investigated the multiaxial yield behavior of 1100-0 aluminum, 70:30 brass, and an overaged 2024 aluminum alloy (2024-T7). It was observed in all three materials that the final direction of the prestrain path strongly influences the distortion of the yield loci. Brass and the 2024-T7 alloy showed more pronounced “kinematic” translation of subsequent yield loci, and the 2024-T7 alloy exhibited a unique behavior—contraction of the yield locus size after plastic deformation. Phillips et al. [11] tested yield surfaces using two different loading paths, stress control and strain control, and discussed the differences in the evolution of the material’s yield surface and loading surface. Wu et al. [12] experimentally determined the initial and subsequent yield surfaces of annealed AISI 304 stainless steel in the axial-torsional stress space and discussed factors influencing the experimental determination of yield surfaces. Zhang et al. [13] used thin-walled tubular specimens of copper to investigate the subsequent yield surfaces under pre-tension, pre-torsion, and pre-proportional tension–torsion by employing the single-sample and multi-sample methods, respectively. The influence of different conditions—including pre-deformation mode, number of test points, test sequence, and specified offset strain (i.e., the component of total strain after subtracting the elastic strain, with the specific qualification that it refers to a small threshold selected for the determination of yielding.)—on the measured subsequent yield surfaces was examined. Differences between the results obtained by the two methods and the concave phenomenon (i.e., a larger negative curvature of the yield surface) observed in the yield surfaces were also discussed. Naghdi et al. [14] utilized thin-walled tubular specimens and an axial-torsion combined loading device to conduct multi-path loading experiments in the tensile-shear stress space. The research indicated that under proportional loading, subsequent yield surfaces primarily exhibited central translation and expansion. Under non-proportional multi-path loading, however, the yield surfaces showed significant distortion and asymmetry, revealing a strong dependence of the material’s hardening behavior on the loading history. Shiratori et al. [15,16] experimentally investigated the stress–strain relationship and subsequent yield surfaces after pre-loading along a strain path with a corner by applying combined axial load, internal pressure, and torque to thin-walled brass specimens. They found that the subsequent yield surfaces were not symmetric about the direction of the corresponding prestress. Phillips et al. [17] presented experimental results concerning the motion of yield surfaces and loading surfaces and the interrelationship between these motions. The study showed that yield surfaces tend to be tangential to loading surfaces, and the plastic strain vector is normal to the yield surface. Khan et al. [18,19] experimentally observed the appearance of a “sharp corner” (i.e., a larger curvature of the yield surface in the direction of pre-loading stress) and a “cross effect” (i.e., a change in the size of the yield surface in the direction perpendicular to the pre-loading direction) on subsequent yield surfaces. After loading in a specific direction, the yield surface significantly deviates from its initial state and does not expand uniformly. The curvature increases in the loading direction, leading to a sharp corner, while it decreases in the opposite direction, making the surface increasingly flattened. Furthermore, changes occur in the yield surface perpendicular to the loading direction, known as the cross effect. All these phenomena result in measured yield surfaces increasingly diverging from the cylindrical surfaces suggested by classical plasticity theory.

Many scholars have employed the single-specimen method to investigate the evolution of subsequent yield surfaces. For instance, Khan et al. [18,19], Phillips et al. [20], and Sung et al. [21] conducted experimental studies on the evolution of subsequent yield surfaces for different materials using this approach. Since the single-specimen method involves measuring multiple yield points along a polygonal path, it inevitably leads to the accumulation of additional plastic deformation. Strictly speaking, the measured yield points do not belong to the same yield surface. Therefore, yielding must be determined using a very small specified offset strain or linear deviation value (e.g., Helling et al. [10] used 5 με, Ellis et al. [22] used 10 με, and Hu et al. [23] used 20 με as the specified offset strain to define yielding). Even so, it remains difficult to verify the validity of the measured yield surface. By contrast, the multi-specimen method employs a separate specimen for each probing direction of the yield surface, and the complete yield surface is constructed from tests on multiple specimens. This approach avoids the accumulation of additional plastic deformation inherent to the single-specimen method, although it may be affected by scatter arising from specimen-to-specimen variability. When the specimens are machined to high precision and exhibit good material uniformity, the multi-specimen method yields more accurate results. According to existing literature, experimental studies on material subsequent yielding using the multi-specimen method are still relatively scarce [13,23,24,25,26,27,28,29,30,31,32,33]. Classical plasticity theory deduces the convexity of yield surfaces based on Drucker’s postulate. However, regardless of whether the single-specimen or multi-specimen method is used, the measured subsequent yield surfaces can exhibit concavity [13,23,26]. This phenomenon has rarely been discussed in depth, and some scholars (e.g., Khan et al. [18,19] and Sung et al. [21]) have avoided addressing the concavity issue in measured yield surfaces by means of selective measurement points or repeated testing.

Ductile cast iron is renowned for its high strength, good toughness, and excellent castability, making it a critical material for key load-bearing components in machinery. Examples include wind turbine main shafts, heavy-duty truck chassis, pipeline systems, and engine crankshafts. Ductile cast iron components often encounter complex and variable loading conditions during actual service. For instance, wind turbine main shafts experience complex combined cyclic loads of torsion and bending. In such scenarios, design based solely on initial yield strength is far from sufficient. It is essential to accurately understand the evolution of the material’s yield surface under complex loading histories to predict its low-cycle fatigue behavior, ratcheting effects, and ultimate multiaxial failure modes. Although considerable research exists on the static strength, fracture toughness, and fatigue performance of ductile cast iron [34,35,36,37,38,39], systematic experimental characterization of its subsequent yield surfaces under multiaxial stress states—especially after undergoing plastic deformation—remains relatively scarce. Existing constitutive models often directly adopt those developed for steels, which creates a significant knowledge gap for modern engineering design that demands high precision and reliability, such as performance-based design and damage tolerance design.

This study focuses on the evolution of subsequent yield surfaces in ductile cast iron. The multi-specimen method is employed to investigate the evolution of subsequent yield surfaces under different pre-deformation levels and unloading positions using ductile cast iron specimens. This research aims both to verify the applicability of classical plasticity theory to ductile cast iron and to advance the scientific understanding of the complex plastic behavior of this important engineering material. Ultimately, this work seeks to provide direct and reliable theoretical foundations and data support for the durability design, life prediction, and safety assessment of related critical components.

## 2. Experimental Materials and Basic Mechanical Properties

**Figure 1:**
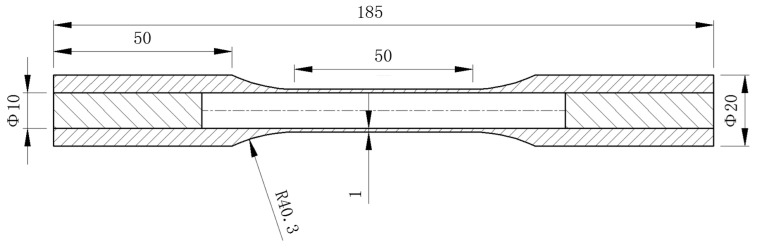
Geometric dimensions of the specimen (mm).

The experimental material used was ductile cast iron QT600-7, with its chemical composition provided in Table 1. The tests employed thin-walled tubular specimens, the geometric dimensions of which are shown in Figure 1.

All tests were conducted on an MTS809 tension–torsion electro-hydraulic servo fatigue testing machine. An axial-torsional MTS632.80F-04 extensometer with a gauge length of 25 mm was used to measure the axial elongation and torsion angle within the gauge section. The torsional sensitivity is 0.015°, and the axial sensitivity is 0.002 mm. The fundamental mechanical parameters of the material, calibrated from the uniaxial tensile curve, are listed in Table 2.

## 3. Experimental Methodology

### 3.1. Initial Yield Surface Testing

**Figure 2:**
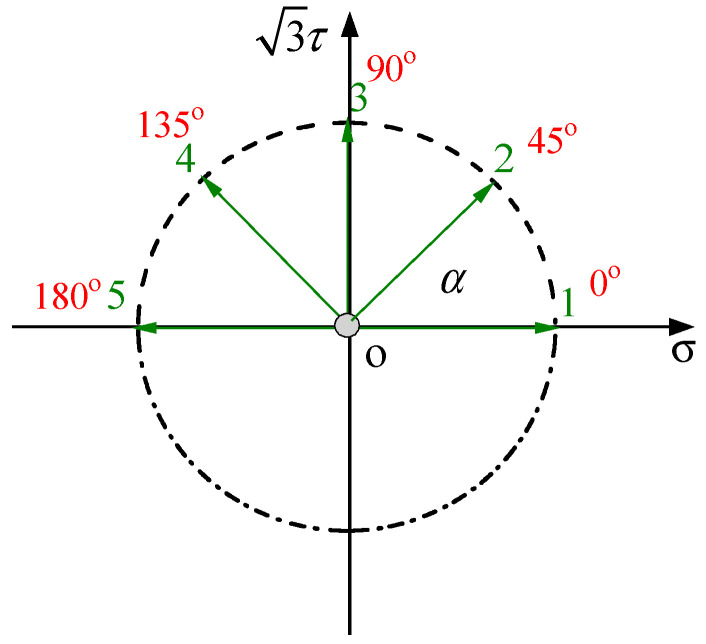
Schematic diagram of the stress-space loading path for the initial yield surface.

The initial yield surface testing employed stress-controlled loading, starting from the origin O. Yield points were probed in the directions of 0°, 45°, 90°, 135°, and 180° by setting different proportional values of axial stress and torsional stress. A schematic diagram of the simulated probing path for the initial yield surface is shown in Figure 2. The multi-specimen method was used for testing the yield surface, where one specimen was dedicated to measuring the yield point in only one specified direction. One yield surface was determined using five specimens, with yield points measured along five different loading directions, and the specimens were not reused.

The determination and identification of the material’s yield point can be achieved by measuring the linear deviation of the loading curve or by detecting the strain offset resulting from residual deformation after unloading. The latter is referred to as the offset strain method, which yields more stable test results and is thus more feasible and reliable. When the material is loaded from a specified initial state point within the elastic range and then unloaded back to the initial point, the strain returns to its prior state without any offset. However, if the elastic range is exceeded, the strain measured after unloading exhibits an offset, denoted as $\sum_{offset}^{strain}$, whose magnitude correlates with the extent to which the elastic range has been exceeded. Therefore, the measured offset strain $\sum_{offset}^{strain}$ can be used as a criterion for identifying material yielding.

### 3.2. Subsequent Yield Surface Testing

The subsequent yield surface test is divided into two stages: pre-loading and subsequent loading. The overall test loading scheme is shown in Table 3.

#### 3.2.1. Pre-Loading

To better investigate the subsequent yield surfaces of ductile cast iron under different pre-loading conditions, three types of pre-loading scenarios were designed in this study: different pre-tension amplitudes, different unloading points, and different pre-loading paths.

**Figure 3:**
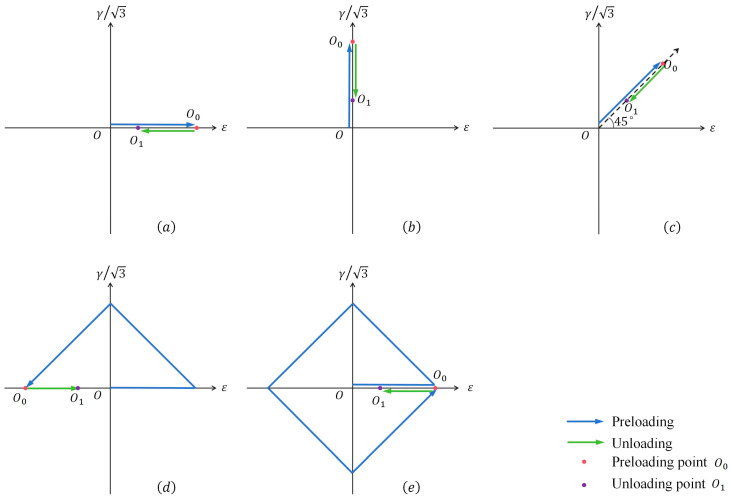
Test loading process and path in strain space for the subsequent yield surface after pre-deformation. (**a**) pre-tension; (**b**) pre-torsion; (**c**) pre-combined tension–torsion; (**d**) pre-triangular; (**e**) pre-diamond.

➀ Different pre-tension amplitudes
  (1) Pre-tension Loading: Specimen loading conditions P1 and P3. Axial tensile pre-loading was performed under strain control. Two pre-tension strain ε amplitudes were set to strains of 0.4% and 0.8%, respectively. In strain space, this pre-loading process corresponds to the stage from O to O_0_ in Figure 3a.
  (2) Unloading: The control mode was switched to stress control. The stress was held at the pre-loading point for 120 s to allow stress relaxation, mitigating the influence of material viscoplasticity and ensuring that the internal stress of the material reached a stable state. Subsequently, the specimen was unloaded to the elastic region ($\sigma =200\;\mathrm{Mpa},\sqrt{3}\tau =0$), as shown in the stage from O_0_ to O_1_ in Figure 3a, where point O_1_ serves as the starting point for subsequent loading.

➁ Different unloading points
  (1) Pre-tension loading: Specimen loading conditions P2 and P3. Axial tensile pre-loading was conducted under strain control, with the pre-tension strain ε amplitude set to 0.8%. In strain space, this corresponds to the stage from O to O_0_ in Figure 3a.
  (2) Unloading: After stress relaxation for 120 s, unloading was initiated. The unloading points O_1_ were set as the elastic unloading point ($\sigma =200\;\mathrm{Mpa},\sqrt{3}\tau =0$) and the stress-free point ($\sigma =0,\;\sqrt{3}\tau =0$), respectively.

➂ Different pre-loading paths
  (1) Five pre-loading paths were established, corresponding to specimen loading conditions P3 to P7, as illustrated in Figure 3. The equivalent strain $\varepsilon^{\mathrm{eq}}$ amplitude for each loading path is listed in Table 3. The equivalent stress and strain mentioned in this paper refer to the von Mises equivalent stress and strain, respectively.
  (2) Unloading: After each of the five pre-loading paths, the specimens were unloaded to the elastic unloading point O_1_ where the equivalent stress $\sigma^{\mathrm{eq}}$ is 200 MPa, as shown in Figure 3a–e, respectively. The specific stress values corresponding to the O_1_ unloading positions are detailed in Table 3.

#### 3.2.2. Subsequent Loading

**Figure 4:**
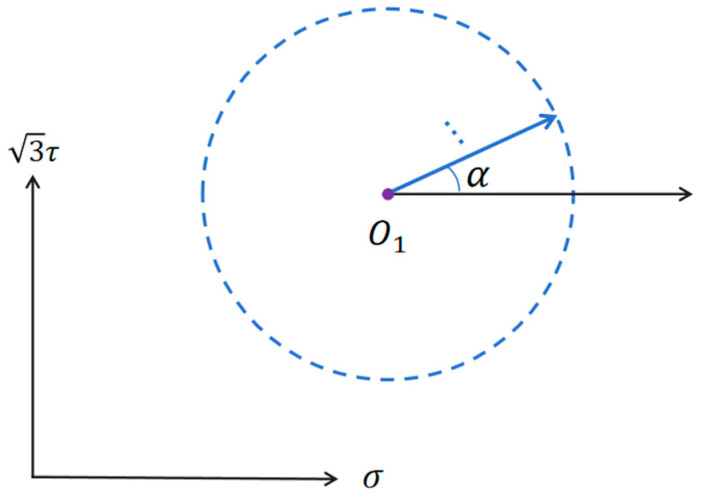
Loading process in stress space for the subsequent yield surface test after pre-deformation.

As shown in Figure 4, subsequent loading initiates from the unloading point O_1_. By setting different ratios of tensile stress σ to shear stress τ, loading is applied along various test directions α, as specified in Table 3. Each test direction α uses a separate specimen for subsequent loading, allowing a series of yield points corresponding to different target offset strains to be obtained. Since the yield surface is symmetrical about the pre-loading axis, yield point testing is conducted only for the angular range of approximately 0° to 180° around this axis. The complete subsequent yield surface can then be obtained through symmetrical extension.

### 3.3. Data and Parameter Calculation

The test data were collected using the software integrated with the MTS fatigue testing machine. The data transmitted via the extensometer and grips of the testing machine are shown in Table 4.

Using tension–torsion loading tests, the yield surface can be represented by its locus in the $\sigma -\sqrt{3\tau }$ stress plane (or space), where each yield point on the surface is defined by the axial stress σ and shear stress τ.

Through tension–torsion loading tests, the axial force *F*, torque *T*, elongation of the gauge section ΔL, and rotation angle θ during loading and unloading can be directly measured. The nominal axial stress σ, shear stress τ, nominal axial strain ε, and shear strain γ can be calculated using the following formulas:

(1) $$\sigma =\frac{4F}{\pi (D^{2}-d^{2})}$$

(2) $$\tau =\frac{16T}{\pi (D^{2}-d^{2})(D+d)}$$

(3) $$\varepsilon =\frac{\Delta L}{L}$$

(4) $$\gamma =\frac{\bar{R}\cdot \;\theta }{L}$$

where *D* and *d* are the outer and inner diameters, respectively, of the gauge section of the thin-walled tubular specimen; $\bar{R}$ and L are the initial mean radius and gauge length of the gauge section, respectively.

The equivalent stress $\sigma_{eq}$ and the equivalent residual plastic strain $\Delta \varepsilon_{eq}^{p}$ relative to the unloading point are calculated using the following formulas:

(5) $$\sigma_{eq}=\sqrt{\sigma^{2}+3\tau^{2}}$$

(6) $$\Delta \varepsilon_{eq}^{p}=\sqrt{{\left(\Delta \varepsilon^{p}\right)}^{2}+\frac{{\left(\Delta \gamma^{p}\right)}^{2}}{3}}$$

(7) $$\Delta \varepsilon^{p}=\Delta \varepsilon -\frac{\Delta \sigma }{E}$$

(8) $$\Delta \gamma^{p}=\Delta \gamma -\frac{\Delta \tau }{G}$$

where *E* and *G* are the elastic modulus and shear modulus of the material, respectively. All the stress and strain increments mentioned above are calculated relative to the unloading point, i.e.,

(9) $$\Delta \varepsilon =\varepsilon -\varepsilon_{O_{1}}$$

(10) $$\Delta \gamma =\gamma -\gamma_{O_{1}}$$

(11) $$\Delta \sigma =\sigma -\sigma_{O_{1}}$$

(12) $$\Delta \tau =\tau -\tau_{O_{1}}$$

For the multi-specimen method, $\varepsilon_{O_{1}}$ and $\gamma_{O_{1}}$ remain constant, $\sigma_{O_{1}}$ and $\tau_{O_{1}}$ remain unchanged at the unloading point.

During the testing process, when the cumulative equivalent residual plastic strain $\Delta \varepsilon_{eq}^{p}$ reaches the specified offset strain $\sum_{offset}^{strain}$ during successive loading, the corresponding axial stress and shear stress can be calculated by Equations (1)–(8). Subsequently, the yield surface (or curve) can be plotted as a series of yield points in the $\sigma -\sqrt{3\tau }$ space.

Let a planar curve be given by an ordered set of points ($\sigma_{i}$,$\sqrt{3}\tau_{i}$) for i = 1, 2, …, *N*. For each interior point i (2 ≤ i ≤ N − 1), the signed curvature $\kappa_{i}$ is estimated using the three consecutive points ($\sigma_{i-1}$,$\sqrt{3}\tau_{i-1}$), ($\sigma_{i}$,$\sqrt{3}\tau_{i}$), and ($\sigma_{i+1}$,$\sqrt{3}\tau_{i+1}$).

Define the two edge vectors emanating from the middle point:

(13) $$v_{i}=\left(\begin{array}{c} \sigma_{i}-\sigma_{i-1}\\ \sqrt{3}\tau_{i}-\sqrt{3}\tau_{i-1} \end{array}\right)\;\;,\;\;\;\;w_{i}=\left(\begin{array}{c} \sigma_{i+1}-\sigma_{i}\\ \sqrt{3}\tau_{i+1}-\sqrt{3}\tau_{i} \end{array}\right)$$

Their lengths (Euclidean distances) are

(14) $$L_{i}^{\left(\mathrm{left}\right)}=∥v_{i}∥=\sqrt{{\left(\sigma_{i}-\sigma_{i-1}\right)}^{2}+{\left(\sqrt{3}\tau_{i}-\sqrt{3}\tau_{i-1}\right)}^{2}}$$

(15) $$L_{i}^{\left(\mathrm{right}\right)}=∥w_{i}∥=\sqrt{{\left(\sigma_{i+1}-\sigma_{i}\right)}^{2}+{\left(\sqrt{3}\tau_{i+1}-\sqrt{3}\tau_{i}\right)}^{2}}$$

The distance between the two outer points is

(16) $$L_{i}^{\left(\mathrm{outer}\right)}=\sqrt{{\left(\sigma_{i+1}-\sigma_{i-1}\right)}^{2}+{\left(\sqrt{3}\tau_{i+1}-\sqrt{3}\tau_{i-1}\right)}^{2}}$$

The signed cross product of $v_{i}$ and $w_{i}$ is

(17) $$C_{i}=v_{i}\times w_{i}=(\sigma_{i}-\sigma_{i-1})(\sqrt{3}\tau_{i+1}-\sqrt{3}\tau_{i})-(\sqrt{3}\tau_{i}-\sqrt{3}\tau_{i-1})(\sigma_{i+1}-\sigma_{i})$$

The sign of $C_{i}$ indicates the turning direction when moving from point i − 1 to i + 1:

$C_{i}$ > 0 means a left turn (locally convex with respect to the forward direction),
$C_{i}$ < 0 means a right turn (concave), and
$C_{i}$ = 0 means the three points are collinear.

The signed curvature at the middle point is computed from the circumscribed circle of the three points:

(18) $$\kappa_{i}=\frac{2C_{i}}{L_{i}^{\left(left\right)}L_{i}^{\left(right\right)}L_{i}^{\left(outer\right)}}$$

The denominator is always positive (provided no two points coincide), so the sign of $\kappa_{i}$ is exactly the sign of $C_{i}$.

If any two successive points are identical (e.g., $L_{i}^{\left(\mathrm{left}\right)}$ = 0 or $L_{i}^{\left(\mathrm{right}\right)}$ = 0), the curvature is undefined. In such cases we set $\kappa_{i}$ = 0 because a repeated point does not contribute to bending.

## 4. Experimental Results and Analysis

First, sharp corners and concavity must be defined based on the curvature of the yield surface. However, the measured yield points are too sparse, leading to significant errors in curvature calculation. Therefore, a cubic spline function is used to interpolate the yield points, and the curvature at these points is then calculated using Equation (18) in both the pre-loading direction and its opposite. The results are presented in Table 5.

The average curvature of the initial yield surface is about 0.003, and the maximum is about 0.008. Therefore, a curvature greater than 0.01 in the same direction as the pre-loading is used as the criterion for a sharp corner. For the direction opposite to the pre-loading, the curvature of the yield surface is negative, indicating a concave shape. The closer the curvature is to zero, the closer the adjacent points are to a straight line, i.e., a flattened state.

### 4.1. Initial Yield Surface

**Figure 5:**
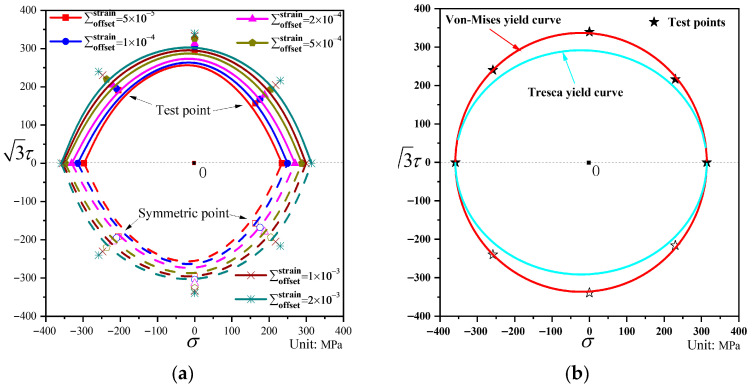
Measured initial yield points. (**a**) Measured initial yield surface under different offset strains; (**b**) Measured yield points and yield locus at an offset strain of 2000 με.

The measured yield points and their fitted curves under different offset strains are shown in Figure 5a. The measured yield point at the offset strain of 2000 με is compared with the Tresca yield locus and the von Mises yield locus, as shown in Figure 5b.

It can be observed from Figure 5a that the yield points corresponding to different offset strains in the same test direction are very close, indicating that the ductile cast iron QT600-7 used in this study exhibits low hardening behavior with a gentle hardening curve, and the stress values corresponding to different offset strains show little variation. The yield surface obtained by fitting the yield points is essentially circular. The initial yield surface was verified against the Tresca and von Mises yield criteria. It can be seen from Figure 5b that the experimentally obtained yield points largely lie on the von Mises yield locus, whereas the deviation from the Tresca yield locus is relatively larger. The yield points in the axial tension and compression directions are close to the Tresca locus, but differ significantly from those obtained under pure torsion and combined tension–torsion loading. This is because the Tresca criterion does not account for the influence of the intermediate principal stress, considering only the maximum and minimum principal stresses, whereas the von Mises criterion incorporates the effects of the maximum, minimum, and intermediate principal stresses. The initial yield surface test demonstrates that the von Mises yield criterion, which considers the intermediate principal stress, aligns better with the experimental results than the Tresca criterion. Through comparative analysis with the yield points, the radii of the von Mises circles that best match the yield points are listed in Table 6. The figures show that the distribution of yield points at larger offset strains correlates more closely with the von Mises circle, while minor deviations exist at smaller offset strains.

The yield points obtained from the initial yield surface test exhibit good agreement with the von Mises yield locus. Therefore, the von Mises circle is adopted as the initial yield surface in the subsequent analysis and will be compared with the subsequent yield surfaces obtained from different loading tests.

### 4.2. Statistical Analysis of Specimen Dispersion

**Figure 6:**
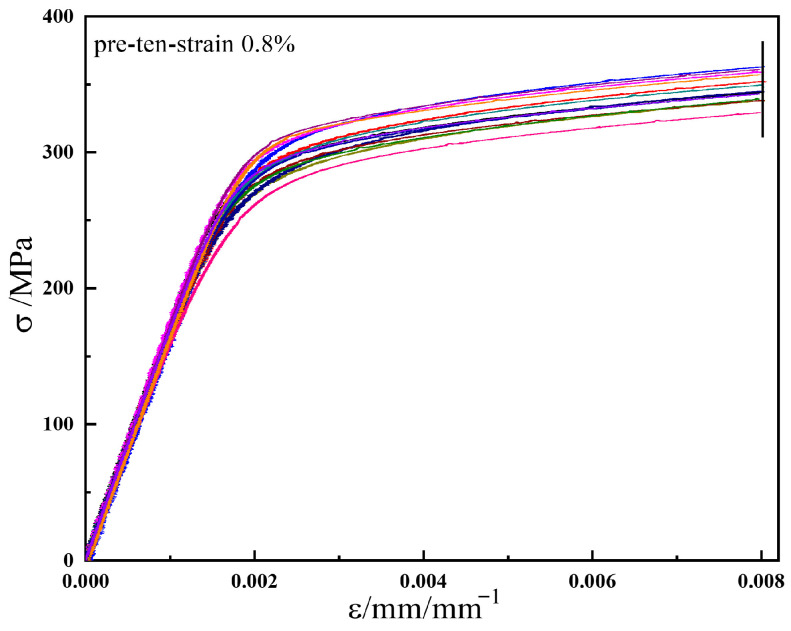
The axial force–axial strain curves of the different specimens.

To evaluate the dispersion among specimens, the elastic modulus and maximum stress from the stress–strain curves during the pre-tension stage (pre-deformation level ε = 0.8%) for the subsequent yield surfaces were compared, as shown in Figure 6. The stress–strain curves of different specimens are represented by different colors in Figure 6. The specimen dispersion was assessed through a statistical analysis of the elastic modulus and maximum stress in the pre-tension stage.

**Figure 7:**
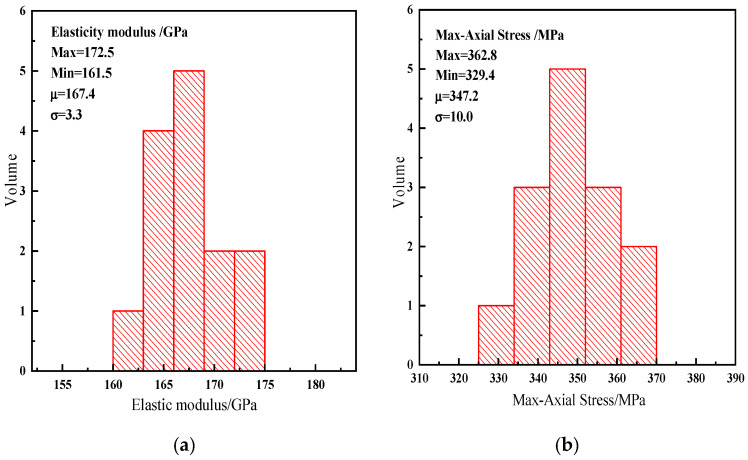
Statistical distribution of elastic modulus and maximum axial force during the pre-tension stage. (**a**) Distribution of Elastic Modulus; (**b**) Distribution of Maximum Axial Force.

Table 7 presents the specific data of the elastic modulus and maximum stress during the pre-tension stage, while Figure 7 shows the statistical distribution diagrams of these two parameters. In Figure 7a, the average elastic modulus of ductile cast iron QT600-7 is 167.4 GPa, with the minimum value (161.5 GPa) showing a maximum deviation of 3.53%. The standard deviation is 3.3, as listed in Table 7. The histogram indicates that the elastic modulus values are clustered around the mean, suggesting a small scatter among specimens and thus good uniformity of the material. In Figure 7b, the average maximum axial stress during the pre-tension stage is 347.2 MPa, with the minimum value (329.4 kN) exhibiting a maximum deviation of 5.13%, and the standard deviation is 10.0. These results demonstrate that, due to the high machining accuracy of the specimens and the excellent material homogeneity, the dispersion among specimens is very small, and its influence on the test results is negligible. Consequently, the experimental results for both the initial and subsequent yield surfaces can be considered reliable.

### 4.3. Subsequent Yield Surfaces

**Figure 8:**
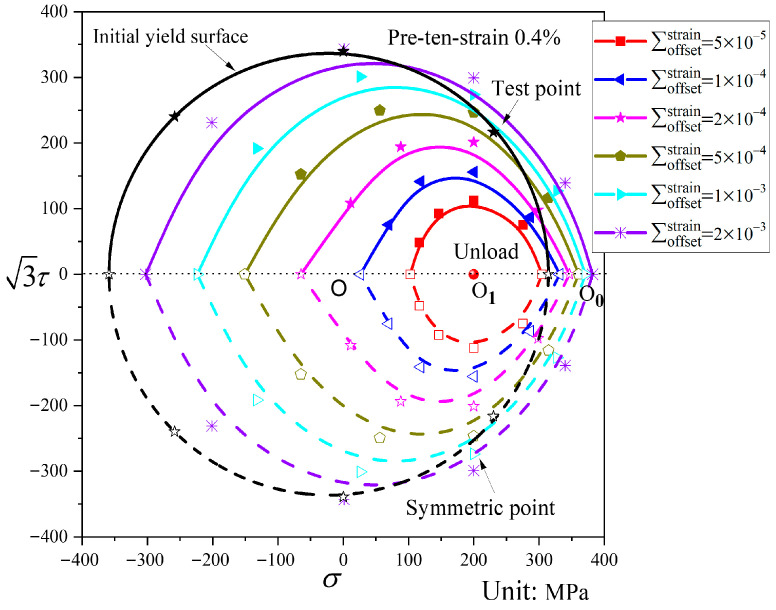
Subsequent yield surfaces for different offset strains after pre-tension to a strain of 0.4% (P1).

Figure 8 shows the subsequent yield surfaces for different offset strains following pre-tension at a strain of 0.4%, along with the initial yield surface corresponding at the offset strain of 2000 με. As shown in Figure 8, for a smaller offset strain following pre-tension to a strain of 0.4%, the subsequent yield surface exhibits a reduced size and shifts more markedly toward the pre-loading direction compared with the initial yield surface. In contrast, the size change in the subsequent yield surface with a larger offset strain is not obvious, and its shift toward the pre-loading direction is less pronounced. The shape variation in the subsequent yield surface after pre-tension to a strain of 0.4% is generally small, remaining close to the initial yield surface and essentially circular.

**Figure 9:**
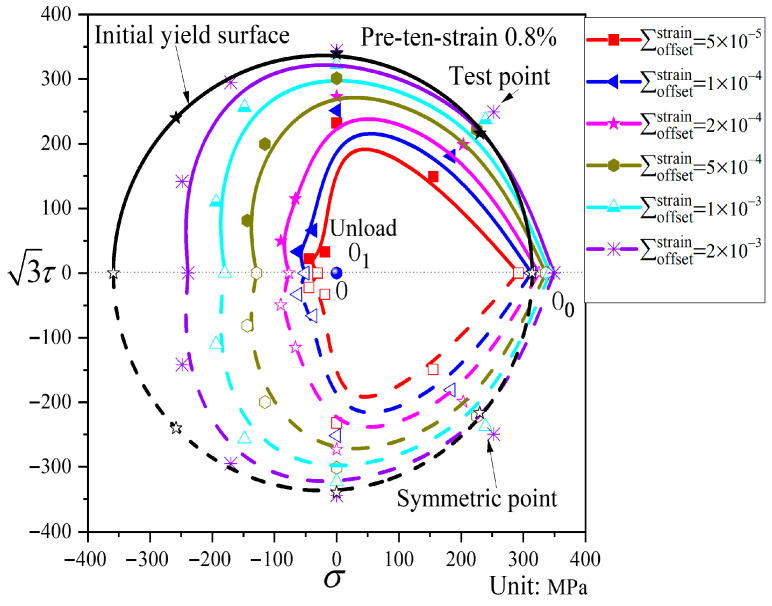
Subsequent yield surfaces corresponding to different offset strains after pre-tension to a strain of 0.8% and unloaded to the stress-free point (P2).

Figure 9 shows the subsequent yield surfaces after pre-tension to a strain of 0.8% and unloading to the stress-free point. Relative to the initial yield surface, the subsequent yield surfaces exhibit considerable distortion but only limited translation. For small offset strains, the subsequent yield surface is markedly distorted, displaying a distinct “sharp corner” in the pre-loading direction and a rather complex deformation morphology in the opposite direction (this complexity arises because after pre-tension and unloading to the stress-free point, the material is no longer in the elastic region; thus, reverse compression leads to yielding, resulting in intricate deformation patterns under small offset strains in the opposite direction of pre-loading). As the offset strain increases, the yield surfaces gradually approach the shape of the initial yield surface, while the overall translation remains relatively small across different offset strains.

**Figure 10:**
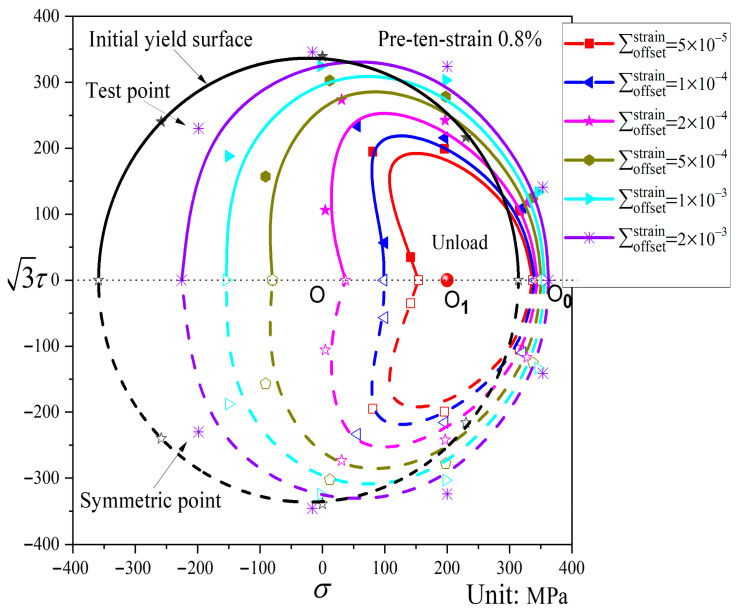
Subsequent yield surfaces corresponding to different offset strains after pre-tension to a strain of 0.8% and unloading to the elastic stage (P3).

As shown in Figure 10, the subsequent yield surfaces after pre-tension to a strain of 0.8% undergo considerable shape changes relative to the initial yield surface. The smaller the offset strain, the more pronounced the distortion of the subsequent yield surface. An inconspicuous “sharp corner” appears in the pre-loading direction, while a clear “concave” feature emerges in the opposite direction. The yield surface shifts noticeably toward the pre-loading direction. As the offset strain increases, the “sharp corner” in the pre-loading direction gradually disappears, the “concave” feature in the opposite direction progressively weakens and eventually flattens, and the translation toward the pre-loading direction becomes less significant.

**Figure 11:**
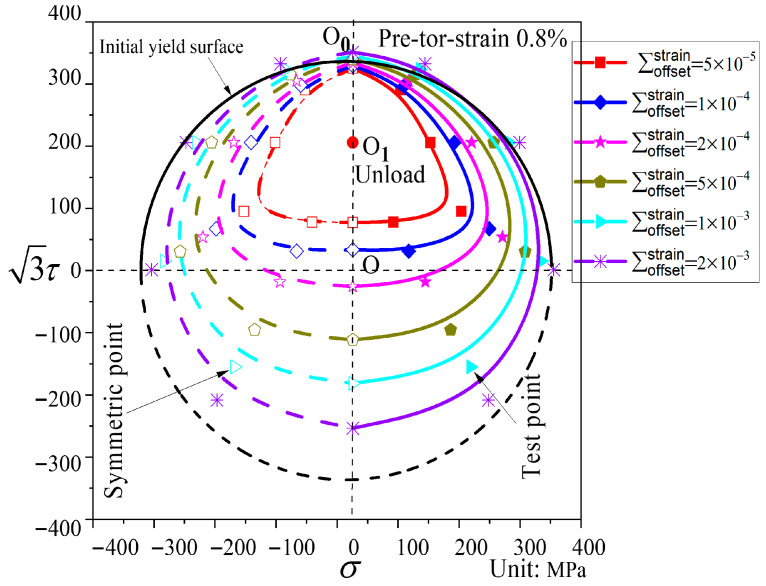
Subsequent yield surfaces corresponding to different offset strains after pre-torsion to a strain of 0.8% (P4).

In Figure 11, relative to the initial yield surface, the subsequent yield surface after pre-torsion exhibits significant translation and distortion when the offset strain is small. For small offset strains, a very pronounced “sharp corner” appears in the pre-loading direction, while the opposite direction shows a flattened state. The yield surface shifts substantially toward the pre-loading direction, and its size is reduced. As the offset strain increases, the yield surface gradually approaches the initial yield surface, and the amount of translation becomes less pronounced.

**Figure 12:**
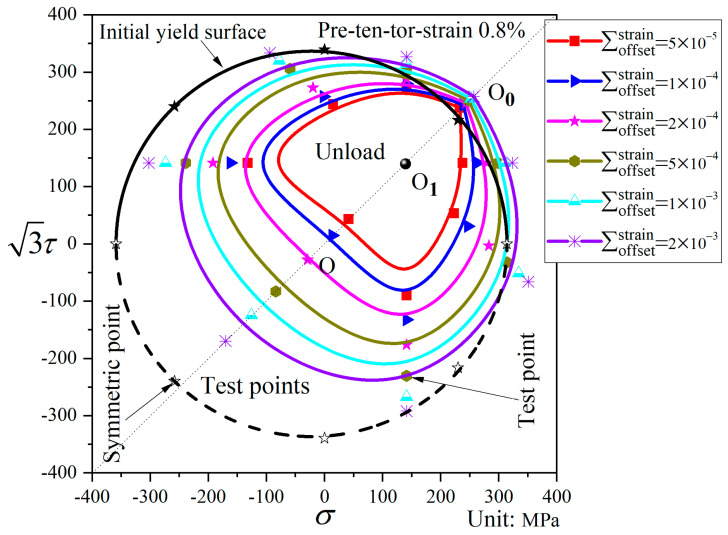
Subsequent yield surfaces for different offset strains after pre-combined tension–torsion to a strain of 0.8% (P5).

In Figure 12, relative to the initial yield surface, the subsequent yield surface after pre-combined tension–torsion exhibits significant shape changes. For small offset strains, a “sharp corner” appears in the pre-loading direction, while a “concave” feature is observed in the opposite direction, accompanied by substantial translation toward the pre-loading direction. As the offset strain increases, the shape of the yield surface gradually approaches that of the initial yield surface, showing a flattened state in the direction opposite to the pre-loading, with minimal translation.

Subsequent yield surfaces after pre-combined tension–torsion were tested within a full 360° range (α = 0°, ±45°, ±90°, ±135°, 180°). As can be seen from the figure, the distribution of the yield points is symmetric about the pre-loading axis, indicating that the yield surface is symmetric with respect to the pre-loading direction. In this study, except for the pre-combined tension–torsion subsequent yield surface, all other initial and subsequent yield surfaces were tested only within a 180° range. Because the subsequent yield surfaces are symmetric about the pre-loading axis, the yield points for the remaining 180° range are obtained by symmetrical extension. Subsequently, the entire yield surface is constructed by curve fitting of the yield points.

**Figure 13:**
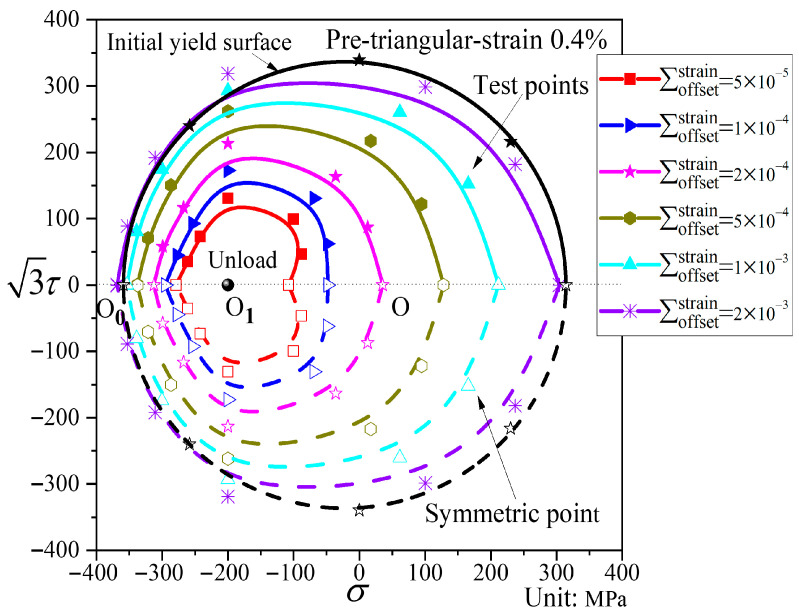
Subsequent yield surfaces corresponding to different offset strains under pre-triangular stress path loading (P6).

In Figure 13, compared with the initial yield surface, the subsequent yield surface under triangular pre-loading exhibits both translation and distortion. The smaller the offset strain, the more pronounced the “sharp corner” that appears in the pre-loading direction, while a relatively clear “concave” feature also emerges in the opposite direction. The size of the yield surface is reduced, and it shifts considerably toward the pre-loading direction. As the offset strain increases, the shape of the yield surface gradually approaches that of the initial yield surface, and the translation toward the pre-loading direction becomes less significant. At larger offset strain $\sum_{offset}^{strain}$ = 2000 με, the yield surface essentially undergoes neither translation nor distortion.

**Figure 14:**
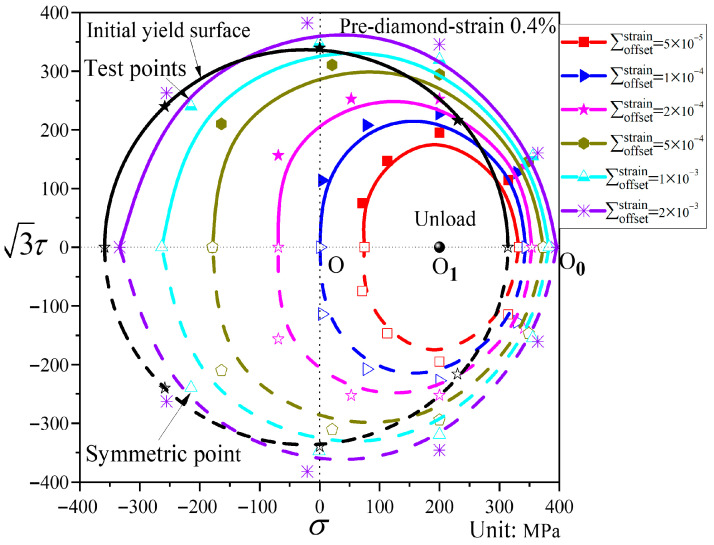
Subsequent yield surfaces for different offset strains under pre-diamond stress path loading (P7).

In Figure 14, compared with the initial yield surface, the subsequent yield surface under diamond pre-loading undergoes little shape change. For small offset strains, the subsequent yield surface shows a flattened state in both the pre-loading direction and its opposite direction, but its size is reduced, presenting an elliptical shape with the major axis oriented along the torsional direction. It shifts substantially toward the pre-loading direction. As the offset strain increases, the shape of the yield surface gradually tends to become circular. At offset strains between 1000 and 2000 με, the subsequent yield surface expands slightly along the torsional direction relative to the initial yield surface.

### 4.4. Factors Influencing the Evolution of Subsequent Yield Surfaces

#### 4.4.1. Influence of Pre-Loading Path on Subsequent Yield Surfaces

Figure 10, Figure 11 and Figure 12 show the subsequent yield surfaces after pre-deformation $\varepsilon^{eq}=0.8\%$ along proportional paths, with the pre-loading paths being pre-tension, pre-torsion, and pre-combined tension–torsion, respectively. Comparison reveals that all three types of yield surfaces shift toward the pre-loading direction, and the amount of shift is essentially similar; in each case, the translation toward the pre-loading direction decreases as the offset strain increases. Regarding the distortion of the yield surfaces, when the offset strain is large, the subsequent yield surfaces under different proportional pre-loading paths all approach the initial yield surface. The difference lies in the behavior at small offset strains: a “sharp corner” appears in the pre-loading direction for all three cases, but the opposite direction exhibits distinct features. For pre-tension and pre-combined tension–torsion, a “concave” phenomenon is observed in the direction opposite to the pre-loading, whereas for pre-torsion, the opposite direction merely becomes flattened.

**Figure 15:**
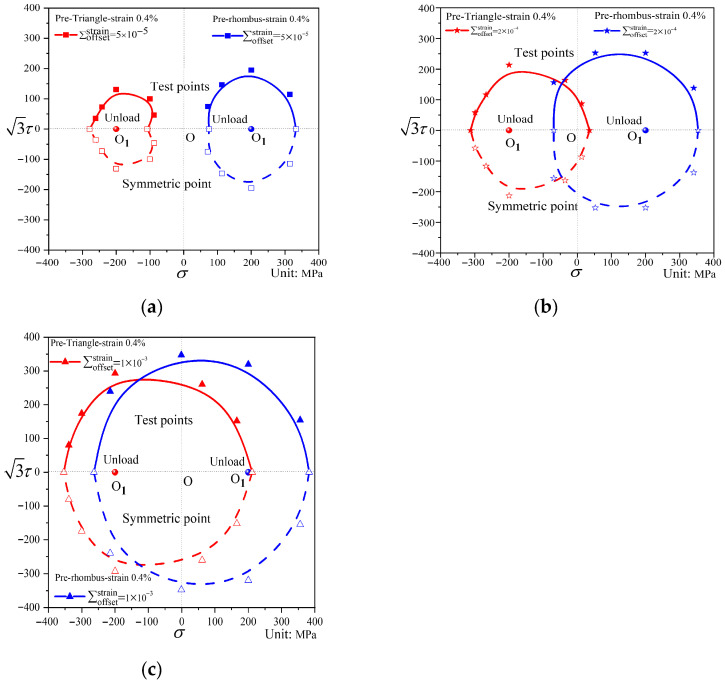
Comparison of subsequent yield surfaces corresponding to different offset strains under pre-triangular and pre-diamond loading. (**a**) $\sum_{offset}^{strain}=50\;\mu \varepsilon$; (**b**) $\sum_{offset}^{strain}=200\;\mu \varepsilon$; (**c**) $\sum_{offset}^{strain}=1000\;\mu \varepsilon$.

Figure 13 and Figure 14 present the subsequent yield surfaces under non-proportional path loading, with the pre-loading paths being triangular and diamond-shaped, respectively. For convenience of comparison, the yield surfaces at the same offset strain under the two paths are displayed in the same coordinate system, as shown in Figure 15. The two paths exhibit differences in both translation and distortion of the yield surfaces: the triangular path shows very little translation toward the pre-loading direction, whereas the diamond path shifts more significantly. At small offset strains, the subsequent yield surface under triangular loading undergoes substantial distortion, displaying distinct “sharp corners” in the pre-loading direction and “concave features” in the opposite direction. In contrast, the subsequent diamond-shaped yield surface takes an elliptical form. When the offset strain is larger, both shapes approach the initial yield surface. Under the same offset strain, the subsequent yield surface after diamond pre-loading expands relative to that after triangular pre-loading.

#### 4.4.2. Influence of Pre-Deformation Level on Subsequent Yield Surfaces

**Figure 16:**
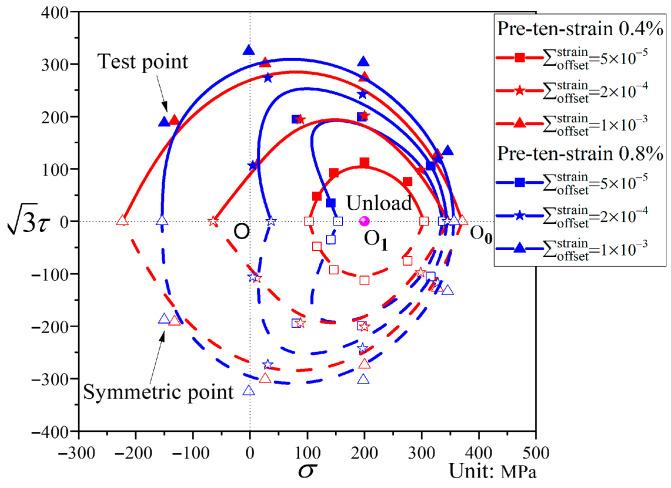
Comparison of subsequent yield surfaces after pre-tension to strains of 0.4% and 0.8% for different offset strains.

A comparison of the subsequent yield surfaces after pre-tension to strains of 0.4% and 0.8% is shown in Figure 16. It can be observed that, when the offset strain is small, the subsequent yield surface after pre-tension to 0.4% strain exhibits little distortion, whereas that after pre-tension to 0.8% strain displays a distinct “sharp corner” in the pre-loading direction and a “concave” feature in the opposite direction. This indicates that the level of pre-deformation influences the distortion of the subsequent yield surface: the greater the pre-deformation, the more pronounced the distortion becomes. For both pre-deformation levels, the smaller the offset strain, the more the yield surface shifts toward the pre-loading direction, and the magnitude of translation is similar. Therefore, the pre-deformation level primarily influences the shape of the subsequent yield surface, with negligible effect on its translation. This phenomenon may be related to the low hardening characteristics of QT600-7.

#### 4.4.3. Influence of Offset Strain on Subsequent Yield Surfaces

Analysis of Figure 8, Figure 9, Figure 10, Figure 11, Figure 12, Figure 13 and Figure 14 reveals that the offset strain has a significant influence on the translation and distortion of subsequent yield surfaces. Regarding the translation of subsequent yield surfaces, the smaller the offset strain, the more the yield surface shifts toward the pre-loading direction. As the offset strain increases, the translation toward the pre-loading direction decreases. With respect to the distortion of subsequent yield surfaces, smaller offset strains lead to more pronounced distortion, manifesting as a “sharp corner” in the pre-loading direction and either “concave features” or a flattened state in the opposite direction. As the offset strain increases, the subsequent yield surface gradually approaches the initial yield surface.

#### 4.4.4. Influence of Unloading Position on Subsequent Yield Surfaces

**Figure 17:**
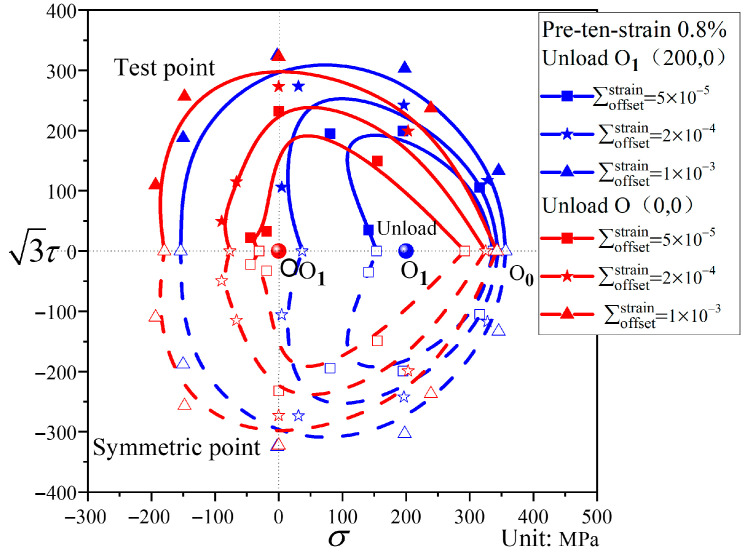
Comparison of subsequent yield surfaces corresponding to three offset strains unloaded to different positions after pre-tension to a strain of 0.8%.

A comparison of the subsequent yield surfaces after pre-tension to a strain of 0.8% and unloading to different unloading points is shown in Figure 17. The pre-deformation level is the same in both cases; the difference lies in the unloading position—one is unloaded to the elastic stage, while the other is unloaded to the stress-free point (where reverse compression leads to yielding). As can be seen from Figure 17, the unloading position has a significant influence on the translation and distortion of the subsequent yield surfaces. Regarding the translation, the subsequent yield surface unloaded to the stress-free point shifts less toward the pre-loading direction, whereas the one unloaded to the elastic stage shifts more. With respect to the distortion, when the offset strain is large, the unloading position has little effect on the distortion of the yield surface. However, when the offset strain is small, a “concave” phenomenon appears in the opposite direction of the pre-loading for the subsequent yield surface unloaded to the elastic stage, while the subsequent yield surface unloaded to the stress-free point exhibits a complex deformation morphology.

### 4.5. Analysis of the Pre-Loading Axis for Subsequent Yield Surfaces

As observed in Figure 8, Figure 9, Figure 10, Figure 11, Figure 12, Figure 13 and Figure 14, the yield points on all subsequent yield surfaces are densely distributed in the pre-loading direction and sparsely distributed in the opposite direction. Therefore, the stress–plastic strain curves for subsequent loading after pre-tension and pre-torsion are analyzed along the pre-loading axis (i.e., the pre-loading direction and its opposite direction) to investigate the reasons for the dense (or sparse) distribution of yield points.

**Figure 18:**
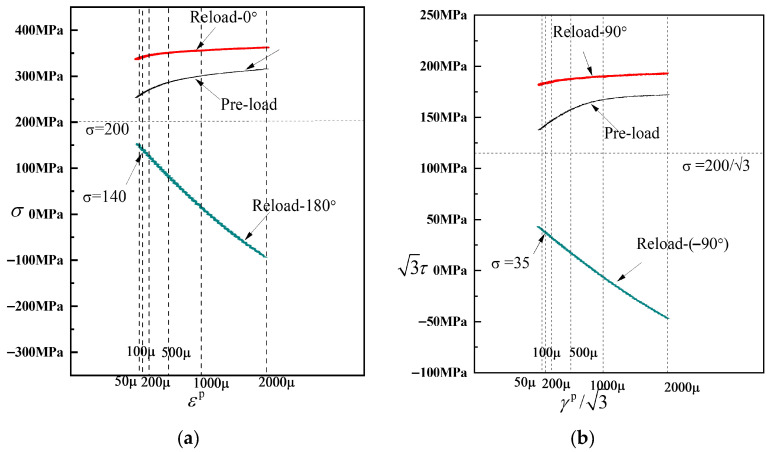
Stress–plastic strain curves for subsequent loading in the same direction as and opposite to the pre-loading direction. (**a**) pre-tension; (**b**) pre-torsion.

Figure 18a shows the stress–plastic strain curves for subsequent loading in the same direction as and opposite to the pre-loading direction after pre-tension to a strain of 0.8% followed by unloading. Similarly, Figure 18b shows the stress–plastic strain curves for subsequent loading in the same direction as and opposite to the pre-loading direction after pre-torsion to a strain of 0.8% followed by unloading. As can be observed from Figure 18a,b, the subsequent loading curve in the same direction as the pre-loading direction is relatively flat, indicating that the hardening stage in the pre-loading direction is weakened after pre-loading. The yield points corresponding to different offset strains on this curve exhibit very small differences in stress, resulting in relatively dense yield points on the subsequent yield surface in the pre-loading direction. In contrast, the subsequent loading curve in the opposite direction to the pre-loading direction is steeper, indicating that the hardening stage in the opposite direction is enhanced after pre-loading. Consequently, the yield points obtained from this curve in the opposite direction are relatively sparse. In addition, the slope of the stress–plastic strain curve in the direction opposite to pre-loading is greater in the 50–500 με range than in the 500–2000 με range, which accounts for the concave or flattened shape observed opposite the pre-loading direction.

## 5. Discussion

The pre-loading paths employed in this study were designed to investigate several factors that influence the evolution of subsequent yield surfaces in ductile cast iron. The three proportional loading paths—pre-tension (P3), pre-torsion (P4), and combined tension–torsion (P5)—correspond to loading directions that form angles of 0°, 90°, and an intermediate orientation relative to the principal stress directions. These three paths are monotonic loading, but actual service loads in engineering structures are often more complex. Therefore, two non-proportional loading paths—triangular (P6) and diamond (P7)—were introduced. In the P6 and P7 pre-loading paths, the principal stress axes undergo rotation. Moreover, P7 exhibits a larger accumulation of plastic deformation. The experimental results show that sharp corners or concave shapes appear at small offset strains under all five paths, indicating that these features are not artifacts of a particular pre-loading path.

By comparing the experimental results of P1 and P3, it can be observed that the distortion of the subsequent yield surface is relatively insignificant under small pre-loading strain amplitude. A possible reason for this phenomenon is that, under pre-loading at small strain amplitudes, the plastic deformation is relatively limited, and the evolution of the subsequent yield surface is primarily driven by dislocation activity on slip systems in a few directions. In contrast, under large strain amplitudes, the microstructure of the metallic material undergoes more substantial changes, which in turn lead to alterations in the shape of the subsequent yield surface. Such microstructural changes may also account for the appearance of sharp corners and concave shapes at small offset strains and their gradual disappearance as the offset strain increases. From a macroscopic perspective, the yield surfaces measured in this study are offset-dependent probing surfaces. The determination of true yield surfaces would require further investigation from a microscopic viewpoint.

In addition, the experimental results obtained in this study are sufficient to qualitatively demonstrate the phenomena of sharp corners and concavity; however, quantitative analysis would require a denser set of probing points and higher-precision experimental equipment.

## 6. Conclusions and Future Research Directions

Based on the analysis of the test results of subsequent yield surfaces, the following conclusions can be drawn:

(1) Compared with the initial yield surface, subsequent yield surfaces shift toward the pre-loading direction, with the degree of distortion varying with offset strain.
(2) The smaller the offset strain, the greater the shift toward the pre-loading direction and the more pronounced the distortion of the subsequent yield surface. As the offset strain increases, both the shift and distortion diminish.
(3) The degree of distortion of the yield surface differs under different pre-loading paths. When the offset strain is small, a “sharp corner” appears in the pre-loading direction, while a “concave” or flattened shape occurs in the opposite direction.
(4) The greater the pre-deformation level, the more severe the distortion of the subsequent yield surface corresponding to small offset strains.
(5) For ductile cast iron QT600-7, when the material is unloaded to the stress-free point after pre-tension loading and then subjected to reverse compression, yielding occurs. Under this condition, the subsequent yield surface corresponding to a small offset strain exhibits a complex deformation morphology in the direction opposite to the pre-loading.

Based on the above results and discussion, further in-depth research will be conducted on the following aspects: (1) adding measurement points to quantitatively analyze the distortion of subsequent yield surfaces under small offset strains and (2) analyzing the intrinsic mechanisms responsible for the distortion of the subsequent yield surface under small offset strains from a microstructural perspective.

## Figures and Tables

**Table 1:** Chemical composition of QT600-7 (%).

Component	C	Si	Mn	P	S	Mg
Quality ratio	3.46	2.60	0.38	0.045	0.03	0.045

**Table 2:** Mechanical properties of ductile cast iron QT600-7.

E (GPa)	$\sigma_{0.2}$ (MPa)	$\sigma_{u}$ (MPa)	$\varepsilon_{f}$
171.56	418.84	630.58	7.85%

**Table 3:** Loading scheme for subsequent yield surface testing.

Code	Pre-Loading Path	Equivalent Pre-Loading Strain $\varepsilon^{\mathrm{eq}}$	Pre-Loading Point O_0_ $\left(\varepsilon ,\gamma /\sqrt{3}\right)$ (MPa)	Unloading Point O_1_ $\left(\sigma ,\sqrt{3}\tau \right)$ (MPa)	Subsequent Loading Direction α (°)
P1	Tension	0.004	(0.004, 0)	(200,0)	0, 45, 90, 120, 150, 180
P2	Tension	0.008	(0.008, 0)	(0,0)	0, 45, 90, 120, 150, 180
P3	Tension	0.008	(0.008, 0)	(200,0)	0, 45, 90, 120, 150, 180
P4	Torsion	0.008	(0, 0.008)	(0,200)	90, 45, 0, −30, −60, −90
P5	Combined tension–torsion	0.008	$(0.008/\sqrt{2},0.008/\sqrt{2})$	$200/\sqrt{2},200/\sqrt{2}$	0, 45, 90, 135, 180, −135, −90, −45
P6	Triangular	0.004	(−0.004. 0)	(−200,0)	0, 22.5, 45, 90, 120, 150, 180
P7	Diamond	0.004	(0.004, 0)	(200,0)	0, 45, 90, 120, 150, 180

**Table 4:** The data exported from MTS.

Axial Displacement (mm)	Axial Force (kN)	Time (s)	Torque (kN · mm)	Axial Strain	Nominal Twist Angle (deg)
d	F	T	M	ε	θ

**Table 5:** The curvature of the subsequent yield surface.

Code	Curvature of the Yield Surface											
	Along Pre-Loading Direction						Opposite to Pre-Loading Direction					
	50 με	100 με	200 με	500 με	1000 με	2000 με	50 με	100 με	200 με	500 με	1000 με	2000 με
P1	0.3318	0.4462	0.3459	0.2167	0.1499	0.1069	0.0167	0.0271	0.0235	0.0105	0.0061	0.0040
P2	0.5620	0.3444	0.2587	0.1729	0.1249	0.0924	−0.0852	−0.0516	−0.0252	−0.0102	−0.0058	−0.0038
P3	0.0772	0.0576	0.0259	0.0054	0.0040	0.0035	−0.0283	0.0059	−0.0126	−0.0044	−0.0024	0.0008
P4	0.2427	0.1936	0.1554	0.0930	0.0585	0.0330	−0.0010	0.0018	0.0003	0.0008	0.0029	0.0001
P5	0.0507	0.0354	0.0110	0.0074	0.0055	0.0044	−0.0057	−0.0029	−0.0006	0.0007	0.0013	0.0020
P6	0.4455	0.2410	0.1304	0.1987	0.1195	0.0464	−0.0670	−0.0164	0.0170	0.1167	0.1052	0.1139
P7	0.0026	0.0017	0.0014	0.0022	0.0022	0.0024	−0.0071	−0.0027	−0.0037	−0.0018	0.0003	0.0015

**Table 6:** The von Mises circle radius of yield surface with different offset strain (Unit: MPa).

Offset Strain	50 με	100 με	200 με	500 με	1000 με	2000 με
von Mises circle radius R	266	282	300	318	327	337

**Table 7:** Elastic Modulus and Maximum Stress During the Pre-tension Stage.

Specimens	Elastic Modulus E (GPa)	Maximum Stress σ (MPa)
1	169.8	343.8
2	168.0	352.1
3	165.0	362.8
4	164.9	350.0
5	172.5	358.8
6	164.0	337.6
7	164.8	344.9
8	166.6	338.0
9	161.5	329.4
10	171.6	339.1
11	166.9	343.0
12	167.4	357.1
13	172.4	361.0
14	168.3	343.3
Mean	167.4	347.2
Standard Deviation	3.3	10.0

## Data Availability

The original contributions presented in this study are included in the article. Further inquiries can be directed to the corresponding author.
